# Postpyloric enteral nutrition in the critically ill child with shock: a prospective observational study

**DOI:** 10.1186/1475-2891-7-6

**Published:** 2008-01-31

**Authors:** Jesús López-Herce, Santiago Mencía, César Sánchez, Maria J Santiago, Amaya Bustinza, Dolores Vigil

**Affiliations:** 1 Pediatric intensive care unit, Hospital General Universitario Gregorio Marañón, Madrid, Spain; 2 Preventive medicine department, Hospital General Universitario Gregorio Marañón, Madrid, Spain

## Abstract

**Background:**

Tolerance to enteral nutrition in the critically ill child with shock has not been studied. The purpose of the study was to analyze the characteristics of enteral nutrition and its tolerance in the critically ill child with shock and to compare this with non-shocked patients.

**Methods:**

A prospective, observational study was performed including critically ill children with shock who received postpyloric enteral nutrition (PEN). The type of nutrition used, its duration, tolerance, and gastrointestinal complications were assessed. The 65 children with shock who received PEN were compared with 461 non-shocked critically ill children who received PEN.

**Results:**

Sixty-five critically ill children with shock, aged between 21 days and 22 years, received PEN. 75.4% of patients with shock received PEN exclusively. The mean duration of the PEN was 25.2 days and the maximum calorie intake was 79.4 kcal/kg/day. Twenty patients with shock (30.7%) presented gastrointestinal complications, 10 (15.4%) abdominal distension and/or excessive gastric residue, 13 (20%) diarrhoea, 1 necrotising enterocolitis, and 1 duodenal perforation due to the postpyloric tube. The frequency of gastrointestinal complications was significantly higher than in the other 461 critically ill children (9.1%). PEN was suspended due to gastrointestinal complications in 6 patients with shock (9.2%). There were 18 deaths among the patients with shock and PEN (27.7%). In only one patient was the death related to complications of the nutrition.

**Conclusion:**

Although most critically ill children with shock can tolerate postpyloric enteral nutrition, the incidence of gastrointestinal complications is higher in this group of patients than in other critically ill children.

## Background

Enteral nutrition is safe and effective in most critically ill children [1,2]. The early initiation of enteral nutrition may preserve mechanical and immunological gut barrier function, stimulating intestinal trophism, and reducing bacterial translocation and the incidence of sepsis and multisystem failure [3]. It has few side effects. However, oral or nasogastric feeding is sometimes poorly tolerated by patients on mechanical ventilation, due to the reduced gastric motility secondary to the administration of drugs or to the disease itself, with the onset of abdominal distension and the accumulation of gastric residues, leading to a risk of pulmonary aspiration [4,5]. Transpyloric enteral nutrition is used in the most severely ill patients, who have a lower tolerance to gastric nutrition, and in patients with deep sedation and muscle relaxation, in both adults [6,7] and children [8-10].

However, enteral nutrition is not prescribed in many patients with shock, or only a low feed volume is administered to keep the bowel active. Enteral nutrition increases splanchnic metabolic demands, which may lead to oxygen and/or energy mismatch when the gut is hypoperfused [11]. Shock leads to a rapid and marked reduction in splanchnic perfusion, altering tolerance to enteral nutrition, and can induce functional and structural gastrointestinal alterations and systemic complications [12]. For this reason, critically ill patients who develop shock are often treated with parenteral nutrition. However, a number of studies have shown that adult patients in the postoperative period of cardiac surgery, with haemodynamic disturbances and/or requiring inotropic support, tolerated enteral nutrition adequately [13,14]. We have found no studies which have prospectively analysed the tolerance and adverse effects of enteral nutrition in children with shock. This has been the objective of the present study.

## Patients and methods

A prospective, observational study was performed which included all the critically ill children admitted to the Paediatric Intensive Care Unit who received postpyloric enteral nutrition (PEN). Patients with shock were compared with the rest of critically ill children. The study was approved by the Institutional Review Board.

Shock was defined as a mean blood pressure > 2 SD below the normal level for age after more than 20 ml/kg of volume infusion and/or dopamine > 15 mcg/kg/min and/or adrenaline > 0.3 mcg/kg/min).

The indications to PEN were children on mechanical ventilation, those with an altered conscious level only responding to noxious stimuli, respiratory failure without mechanical ventilation, children at risk of aspiration, and in those who did not tolerate gastric nutrition. The postpyloric tube was inserted by the nursing staff following a protocolised method, by blind insertion or with placement of the patient in a lateral decubitus position, with air insufflation [15]. Confirmation of the position of the tube was initially performed by aspiration and measurement of the pH (it was considered that the tip of the tube was probably in the duodenum if the pH of the aspirate was equal to or higher than 6), and this was subsequently confirmed radiologically. All the tubes were situated between the 1^st ^and 4^th ^portions of the duodenum. A second tube was inserted via the same nasal orifice for drainage of the gastric contents and for measurement of the gastric residue every 3–4 hours.

The type of nutrition administered depended on the age of the patient: in children under 2–3 years, an infant formula was administered (700 kcal/L, × 18 g protein/L); this was substituted by protein hydrolysate in patients with milk-protein intolerance or a suspicion of intestinal damage. Calorie supplements in the form of dextrin-maltose, medium chain triglycerides, or cereals were added in some patients. In children over 2–3 years of age, isocaloric (1.2 kcal/ml), normoproteic (26 g proteins/L) paediatric liquid formulae were administered. The alimentation was started at a rate of 0.5–1 ml/kg per hour, with increases of 0.5–1 ml/kg every 3–4 hours if the gastric residue was less than 25% of the volume administered, until a calorie intake of 60–100 kcal/100 kcal metabolised/day according to the Holiday formula, was achieved.

The following data were gathered prospectively: age, sex, weight, diagnosis, surgery, previous parenteral nutrition and its duration, indications for PEN, duration of admission before starting at PEN, maximum volume and calories administered, duration of the PEN, indications for withdrawal, and subsequent type of nutrition. The doses of vasoactive drugs, sedatives, and muscle relaxants administered during the PEN, the use of mechanical ventilation and its duration, altered liver function (defined as an elevation of the AST to more than twice the normal value or of the bilirubin above 2 mg/dl), and nosocomial pneumonia after starting the PEN (defined according to CDC criteria), were also recorded. The complications of enteral nutrition analysed were: significant abdominal distension, residues of the nutrition in the gastric aspirate with a volume greater than fifty percent of the volume administered in the previous 4 hours, diarrhoea, and necrotising enterocolitis (defined by abdominal distension, intestinal haemorrhage, and ultrasound and radiological findings). Failure of the enteral nutrition was considered to have occurred when complications secondary to the nutrition developed which required its interruption.

The characteristics of the nutrition were compared between the patients with shock and the remainder of critically ill children who received PEN during the study period. The statistical analysis was performed using the SPSS version 12 statistical programme, expressing quantitative variables as means and standard deviations and qualitative variables as percentages. Uni- or bivariate analyses were used to study statistical associations. The Chi-square test was used for the analysis of qualitative variables and Fisher's exact test for quantitative variables when n was less than 20 or when any theoretical value was less than 5. Student's t test was used to compare quantitative variables between independent groups. Significance was taken as p < 0.05.

## Results

Postpyloric enteral nutrition was administered to 526 critically ill children, 65 (12.3%) of whom presented shock.

Patients with shock had a mean (SD) age of 37.6 (54.4) months (range 21 days-22 years) and weight of 14.6 (14.9) kg (range 2.8–70 kg). Thirty-three children (50.8%) in this group were under 1 year of age and 44 patients (67.6%) were male. The patients' diagnoses are summarised in Table 1. The indication for PEN was mechanical ventilation in 64 patients (98.5%) and intolerance to gastric nutrition in 1 (1.5%).

**Table 1:** Diagnoses of patients with shock

**Diagnosis**	**Number and percentage of patients**
**Postoperative cardiac surgery**	43 (66.1%)
**Respiratory insufficiency**	8 (12.3%)
**Other medical diagnoses**	12 (18.4%)
**Other surgery**	2 (3%)
**Total**	65

A comparison of the characteristics of the children with shock and the other critically ill patients who received PEN is presented in Table 2. The children with shock had a significantly higher age and weight than the other critically ill children who received PEN. A significantly higher percentage of patients with shock required dopamine, adrenaline, and milrinone, and the dose of adrenaline and dopamine was also significantly higher in these children than in the other patients (Table 2). The percentage of patients with shock requiring continuous infusions of sedatives (midazolam and fentanyl) and muscle relaxants (vecuronium) was significantly higher than in the other critically ill children. The doses of midazolam and fentanyl were also significantly higher in children with shock.

**Table 2:** Comparison of the clinical characteristics of the children with shock and the other critically ill patients who received postpyloric nutrition

	**Shock**	**Other patients**	**p**
Number of patients	65	461	
Age (months) (median, range)	12 (0.7–264)	5 (0.1–228)	**.020**
Weight (kg) (median, range)	8.8 (2.5–70)	5.3 (2.1–70)	**.0001**
Sex (male/female)	44/21 (2.1/1)	248/213 (1.1/1)	**.045**
Cardiac surgery	44 (67.7%)	330 (71.6%)	.559
Dopamine (median, range)	62 (95.4%) 10 (3–50)	307 (66.6%) 5 (0.5–20)	**.0001** **.0001**
Adrenaline (median, range)	49 (75.4%) 0.3 (0.02–5)	71 (15.4%) 0.2 (0.04–0.3)	**.0001** **.001**
Milrinone (median, range)	45 (69.2%) 0.7 (0.4–0.8)	206 (44.7%) 0.5 (0.5–1)	**.0001** .246
Acute renal failure	26 (40%)	27 (5.9%)	**.0001**
Hepatic disturbances	3 (4.8%)	6 (1.3%)	.081
Nosocomial pneumonia	9 (15.5%)	38 (8.9%)	.152
Mortality	18 (27.7%)	32 (6.9%)	**.0001**
Midazolam (median, range)	64 (98.5%) 7 (2–16)	384 (83.3%) 4 (0.5–20)	**.0001** **.0001**
Fentanyl (median, range)	64 (98.5%) 6.5 (1–14)	366 (79.4%) 4 (1–25)	**.0001** **.0001**
Vecuronium (median, range)	51 (78.5%) 0.1 (0.1–0.25)	179 (38.8%) 0.1 (0.1–0.3)	**.0001** .660

Maximum dose of drugs during nutrition. Units: mcg/kg/min (dopamine, adrenaline, milrinone, midazolam); mcg/kg/h (fentanyl, vecuronium)

The children with shock presented a significantly higher incidence of acute renal failure than the other of children. Hepatic alterations were also more common in this group, though the difference did not reach statistical significance. The mortality among patients with shock was higher than in the other children (Table 2).

The characteristics of the nutrition are presented in Table 3. Parenteral nutrition was administered to 21.5% of children with shock prior to starting the enteral nutrition. This percentage of children receiving parenteral nutrition prior to the PEN and the duration of this nutrition were similar in the two groups of patients. The time of starting the PEN and the percentage of patients in which the PEN was started within the first 48 hours after the patient's admission to the PICU did not differ significantly between the children with shock and the other critically ill children.

**Table 3:** Comparison of the characteristics of nutrition between the children with shock and the other critically ill patients who received postpyloric nutrition

	**SHOCK**	**Other patients**	**p**
Number of patients	65	461	
Parenteral nutrition			
Patients (number, %) (median, range)	14 (21.5%) 4 (1–30)	80 (17.4%) 6 (0–99)	.391 .956
Days before TEN (median, range)	2 (0.1–28)	2 (0.4–90)	.431
Initiation of the TEN within the first 48 h after admission	44 (67.6%)	284 (61.6%)	.275
Calorie intake in the first 24 h of TEN (kcal/100 kcal metabolised/day) (median, range)	44.6 (20–79)	48.5 (27.5–107)	.373
Maximum calorie intake (kcal/100 kcal metabolised/day) (median, range)	83 (15–121)	84 (15–162)	.106
TEN duration (days) (median, range)	13 (0.4–240)	7 (0.5–140)	**.020**
Gastrointestinal complications	20 (30.7%)	42 (9.1%)	**.0001**
Abdominal distension/excessive gastric residue	10 (15.4%)	23 (5%)	**.004**
Diarrhoea	13 (20%)	21 (4.6%)	**.0001**
Necrotising enterocolitis	1 (1.5%)	2 (0.4%)	.432
Duodenal perforation	1 (1.5%)	0	-
Definitive suspension of TEN	6 (9.2%)	5 (1%)	**.0001**

The calorie intake received on the first day of PEN was lower in the children with shock than in the other children but the differences were not statistically significant. There were no differences between the two groups regarding the maximum calorie intake achieved. The duration of the PEN was significantly longer in the children with shock than in the other children (Table 3).

Thirty patients with shock (30.7%) presented gastrointestinal complications during PEN. A significantly higher rate that in the other critically ill children. Specifically, the incidence of abdominal distension and/or gastric residues and the incidence of diarrhoea in the children with shock were significantly higher than in the remainder of the patients (Table 3). No relationship was found between the incidence of digestive tract complications and age, weight, diagnosis, early (first 48 hours) or late administration of the PEN, volume of nutrition, or the calories administered. Definitive withdrawal of the nutrition due to digestive tract complications (duodenal perforation caused by the transpyloric tube, necrotising enterocolitis, gastrointestinal bleeding, diarrhoea, or abdominal distension) was only necessary in 6 children. Death was related to a mechanical complication of the nutrition (duodenal perforation) in 1 patient.

## Discussion

Few studies have evaluated the safety and efficacy of enteral nutrition in patients with shock [13,14,16]. Our study shows that children with shock can be fed by enteral nutrition although the incidence of complications is higher than in other critically ill children. However, the heterogeneity of the population studied, with a wide age range and very diverse diagnoses, is a limitation as it complicates the analysis of the results. In humans, feeding produces an increase in cardiac output and vasodilatation of the mesenteric arteries, maintaining the balance between oxygen delivery and consumption. However, splanchnic oxygen delivery is reduced in shock while splanchnic oxygen consumption remains unaltered [14]. In this situation, feeding can exacerbate the altered oxygen balance, leading to gastrointestinal complications and, on rare occasions, small bowel necrosis [17-20]. However, Rokyta et al showed that a low-dose post-pyloric enteral nutrition in septic patients led to a hyperaemic systemic and hepatosplanchnic response with no alteration of energy balance or oxygen kinetics. The increase in total hepatosplanchnic blood flow was proportional to the increase in the cardiac index [21]. Similar effects were found by Revelly et al in cardiac patients [13].

Patients with shock present other risk factors that could impair enteral tolerance. First, they require the administration of high doses of vasoactive drugs. Adrenaline and high doses of dopamine can reduce intestinal perfusion and impair the tolerance to nutrition. However, if adrenaline and dopamine increase cardiac output, splanchnic perfusion could be improved. King et al, in a retrospective study on 55 critically ill children who received inotropic drugs, found that many patients tolerate enteral nutrition well [22]. Our experience supports this finding [23]. Berger et al found that enteral nutrition was well tolerated in adults with haemodynamic failure after cardiac surgery [14]. However, enteral nutrient delivery was significantly negatively related to the dose of dopamine and noradrenaline [14]. Probably, the effect of vasoactive drugs will depend on the dose and the haemodynamic situation in each patient.

An additional aspect is that children with shock have a higher incidence of acute renal failure and mortality than other critically ill children [23]. Critically ill patients with acute renal failure can tolerate enteral nutrition although the incidence of gastrointestinal complications is higher [24,25]. Finally, bowel motility is decreased in critically ill patients [26] and may be further reduced by high doses of sedatives and muscle relaxants, impairing enteral tolerance. These drugs are administered to patients with shock more frequently and at higher doses than in other critically ill children; despite this, most children with shock in our study presented an adequate tolerance to postpyloric nutrition.

In the majority of our patients, shock did not delay the initiation of enteral nutrition. Berger et al found that enteral nutrition is possible in the first postoperative week after cardiac surgery in adults [14]. However, in that study enteral, nutrition provided an insufficient energy delivery and the patients required additional parenteral nutrition [14]. In our study, the energy delivery administered on the first day of nutrition in patients with shock was lower than in the other children, though there were no significant differences in the maximum calorie intake achieved in the two groups. There are no well planned prospective studies studies that have analysed whether children in shock require the same calorie intake as other critically ill children.

The incidence of abdominal distension, vomiting, and an excessive gastric residue in our children was of 15%. The frequency varies between 20% and 70% in critically ill adults receiving enteral nutrition [4,5]. The presence of an excessive gastric residue and abdominal distension are due to the existence of gastrointestinal paresis with a slowing of intestinal transit. The higher doses of dopamine, sedatives, and muscle relaxants used in our children with shock may be partly responsible for this complication.

The incidence of diarrhoea was similar to that found in the critically ill adults [4,5], but was significantly higher than in the other critically ill children. Shock produces diarrhoea because it impairs small bowel function and permeability. However, diarrhoea in our patients was generally mild and improved after modification of the diet.

Definitive withdrawal of the PEN due to digestive tract complications was required in only 6 patients with shock. Only 2 severe gastrointestinal complications occurred. One patient suffered duodenal perforation due the insertion of the transpyloric tube and other infant developed necrotizing enterocolitis.

## Conclusion

We conclude that most children with shock can receive transpyloric enteral nutrition, although the incidence of digestive tract complications is higher than in other critically ill patients. For this reason, enteral nutrition must be used with caution in patients with shock. Physicians must monitor the patients closely for the onset of gastrointestinal complications (abdominal distension, excessive gastric residue, bloody diarrhoea, or dilated bowel loops or intramural gas on radiographic studies). If gastrointestinal complications develop and do not improve with a reduction or modification or the diet, enteral nutrition must be suspended and substituted by parenteral nutrition.

## Competing interests

The author(s) declare that they have no competing interests.

## Authors' contributions

JL-H: conceived the study and participated in the design, data collection and analysis, and drafting of the manuscript.

SM, CS, MJS, and AB participated in the design, data collection and analysis, and drafting of the manuscript.

DV participated in the design of the study and performed the statistical analysis.

All authors read and approved the final manuscript.
